# Characterization of the 3'UTR of the *BTD* gene and identification of regulatory elements and microRNAs

**DOI:** 10.1590/1678-4685-GMB-2020-0432

**Published:** 2022-02-14

**Authors:** Gerda Cristal Villalba Silva, Taciane Borsatto, Ida Vanessa Doederlein Schwartz, Fernanda Sperb-Ludwig

**Affiliations:** 1 Universidade Federal do Rio Grande do Sul, Programa de Pós-Graduação em Genética e Biologia Molecular, Porto Alegre, RS, Brazil. Universidade Federal do Rio Grande do Sul Programa de Pós-Graduação em Genética e Biologia Molecular Porto Alegre RS Brazil; 2 Hospital de Clínicas de Porto Alegre, Centro de Pesquisa Experimental, Laboratório BRAIN, Porto Alegre, RS, Brazil. Hospital de Clínicas de Porto Alegre Centro de Pesquisa Experimental Laboratório BRAIN Porto Alegre RS Brazil; 3 Hospital de Clínicas de Porto Alegre, Porto Alegre, RS, Brazil. Hospital de Clínicas de Porto Alegre Porto Alegre RS Brazil; 4 Universidade Federal do Rio Grande do Sul, Departamento de Genética, Porto Alegre, RS, Brazil. Universidade Federal do Rio Grande do Sul Departamento de Genética Porto Alegre RS Brazil

**Keywords:** 3′UTR, genetic variants, miRNAs, AU-rich elements, biotinidase

## Abstract

Reduced biotinidase activity is associated with a spectrum of deficiency ranging from total deficiency to heterozygous levels, a finding that is not always explained by the pathogenic variants observed in the *BTD* gene. The investigation of miRNAs, regulatory elements and variants in the 3’UTR region may present relevance in understanding the genotype-phenotype association. The aims of the study were to characterize the regulatory elements of the 3’UTR of the *BTD* gene and identify variants and miRNAs which may explain the discrepancies observed between genotype and biochemical phenotype. We evaluated 92 individuals with reduced biotinidase activity (level of heterozygotes = 33, borderline = 35, partial DB = 20 or total DB= 4) with previously determined *BTD* genotype. The 3’UTR of the *BTD* gene was Sanger sequenced. *In silico* analysis was performed to identify miRNAs and regulatory elements. No variants were found in the 3’UTR. We found 97 possible miRNAs associated with the *BTD* gene, 49 predicted miRNAs involved in the alanine, biotin, citrate and pyruvate metabolic pathways and 5 genes involved in biotin metabolism. Six AU-rich elements were found. Our data suggest variants in the 3'UTR of *BTD* do not explain the genotype-phenotype discrepancies found in Brazilian individuals with reduced biotinidase.

## Introduction

The enzyme biotinidase (EC 3.5.1.12), encoded by the *BTD* gene, catalyzes the cleavage of biocytin into the vitamin biotin, which acts as a cofactor for several carboxylases, such as pyruvate carboxylase, propionyl-CoA carboxylase, 3-methylcrotonyl-CoA carboxylase, and acetyl-CoA carboxylases 1 (alpha) and 2 (beta) (Wolf, 2001). 

**Figure 1 -:**
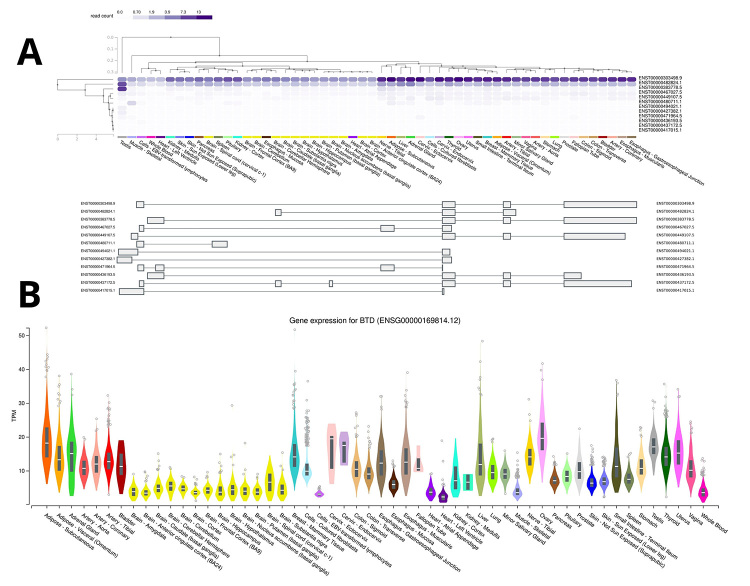
*BTD* gene expression. A: Isoforms in human tissues. B: Expression pattern in several tissues and organs. Adapted from GTEx (https://www.gtexportal.org/home/).

The *BTD* gene is composed by four exons, and its 3′UTR has 331 bp (ENST00000383778.5). The corresponding mRNA has two potential start codons (AUG) (Stanley et al., 2004, Pindolia et al., 2010). There are 17 different 3’UTR lengths with sizes ranging from 77 to 8226 pb, variable according to the transcript. The *BTD* gene has a constitutive expression pattern and healthy individuals present expression between 0.5 and 1.5 log10 transcripts per million (Figure 1). The three-dimensional structure of biotinidase as predicted by *in silico* modeling consists of two domains (Pindolia *et al.*, 2007). 

Biotinidase deficiency (BD) is a metabolic disease, inherited in an autosomal recessive pattern, disabling the body to assimilate biotin from the diet and inhibiting biotin recycling (Baumgartner and Suormala, 2012). If not treated early, BD may lead to neurological and dermatological disorders (Wastell et al., 1988). BD may be total (activity <10% of normal) or partial (10-30%). There is an association between certain genotypes and the observed biochemical phenotype (total or partial), but in some patients, genotype and phenotype are mismatched. According to previous studies by our group, the association between the expected biochemical phenotype (according to genotype) and the actual biochemical phenotype occurs in 68.5% of cases, and variants in the 5′UTR of *BTD* do not seem to explain the variations found (Borsatto et al., 2014, 2017, 2019). Low activity of carboxylases can be found in BD and in Multiple Carboxylase Deficiency, a different disease caused by biallelic pathogenic variations in the *HLCS* gene, which encode the holocarboxylase synthetase enzyme (EC 6.3.4.10).

The aim of this study was to characterize the 3′UTR of the *BTD* gene in individuals with reduced biotinidase activity previously described by our group (Borsatto et al., 2014, 2017, 2019), and to identify which regulatory elements could influence the expression of biotinidase. 

## Material and Methods

The study was approved by the Research Ethics Committee of Hospital de Clínicas de Porto Alegre (n° 16-0480 and 12-0186), Brazil, and the subjects consented to participate by signing the Informed Consent Form.

Ninety-two individuals with reduced biotinidase activity were included: 33 with heterozygous level; 19 with borderline partial/heterozygous; 16 with borderline heterozygous/normal; 20 with partial deficiency; and 4 with total deficiency. These patients had the exons, exon-intron junctions, and 5′UTR of *BTD* previously sequenced, and were described by Borsatto et al. (2014) and Borsatto *et al.* (2017). The genotype and biochemical profile of the cohort is shown in Table 1, and details regarding the classification of the biochemical phenotype and *BTD* sequencing can be found in Borsatto *et al.* (2014) and Borsatto *et al.* (2017). Eighteen individuals had an inconsistent genotype-biochemical phenotype association (1-6, 24-33, 86, 87 - Table 1).

**Table 1 -:** Genetic and biochemical profile of patients with reduced biotinidase activity included in the characterization of the 3’UTR.

Patient	Allele 1	Allele 2	Expected BD according to genotype	Biotinidase activity (nmol/min/mL)	Type of BD according to enzyme activity	Reference
1^#^	c.1330G>C (p.Asp444His)	c.[595C>A;1413T>C] ( p.Val199Met / p.Cys471Cys)	Partial	2.8	Hz	Borsatto et al. (2014)
2^#^	c.[1330G>C;643C>T]^*^	p.Asp444His / p.Leu215Phe^*^	Partial	2.4	Hz	Borsatto et al. (2014)
3^#^	c.1330G>C (p.Asp444His)	c.511G>A (p.Ala171Thr)	Partial	2.5	Hz	Borsatto et al. (2014)
4^#^	c.1330G>C (p.Asp444His)	c.755A>G (p.Asp252Gly)	Partial	2.4	Hz	Borsatto et al. (2014)
5^#^	c.1330G>C (p.Asp444His)	c.1629C>A (p.Asp543Glu)	Partial	2.5	Hz	Borsatto et al. (2017)
6^#^	c.1330G>C (p.Asp444His)	c.755A>G (p.Asp252Gly)	Partial	3.03	Hz	Borsatto et al. (2017)
7	c.[1330G>C;1629C>A]^*^	p.Asp444Hisp / Asp543Glu^*^	Partial / Hz	2.6	Hz	Borsatto et al. (2014)
8	c.[1330G>C;511G>A] (p.Asp444His / p.Ala171Thr)	c.1413T>C (p.Cys471Cys)	Hz	3.3	Hz	Borsatto et al. (2014)
9	c.1330G>C (p.Asp444His)	c.1330G>C (p.Asp444His)	Hz	3.3	Hz	Borsatto et al. (2014)
10	c.1330G>C (p.Asp444His)	c.1330G>C (p.Asp444His)	Hz	4.6	Hz	Borsatto et al. (2017)
11	c.1330G>C (p.Asp444His)	c.1330G>C (p.Asp444His)	Hz	3.2	Hz	Borsatto et al. (2017)
12	c.1330G>C (p.Asp444His)	c.1330G>C (p.Asp444His)	Hz	3.0	Hz	Borsatto et al. (2017)
13	c.1330G>C (p.Asp444His)	c.1330G>C (p.Asp444His)	Hz	3.0	Hz	Borsatto et al. (2017)
14	c.1330G>C (p.Asp444His)	c.1330G>C (p.Asp444His)	Hz	2.8	Hz	Borsatto et al. (2014)
15	c.1330G>C (p.Asp444His)	c.1330G>C (p.Asp444His)	Hz	2.6	Hz	Borsatto et al. (2017)
16	c.1330G>C (p.Asp444His)	c.1330G>C (p.Asp444His)	Hz	3.7	Hz	Borsatto et al. (2014)
17	c.1368A>C (p.Gln456His)	WT	Hz	2.8	Hz	Borsatto et al. (2017)
18	c.1413T>C (p.Tyr494Cys)	c.1629C>A (p.Cys471Cys)	Hz	4.0	Hz	Borsatto et al. (2017)
19	c.643C>T (p.Leu215Phe)	WT	Hz	3.4	Hz	Borsatto et al. (2017)
20	c.1595C>T (p.Thr532Met)	WT	Hz	2.9	Hz	Borsatto et al. (2017)
21	c.1595C>T (p.Thr532Met)	WT	Hz	2.9	Hz	Borsatto et al. (2014)
22	c.364A>G (p.Arg122Gly)	WT	Hz	3.8	Hz	Borsatto et al. (2014)
23	c.[595C>A;1413T>C] (p.Val199Met / p.Cys471Cys)	WT	Hz	3.6	Hz	Borsatto et al. (2017)
24^#^	WT	WT	Normal	2.6	Hz	Borsatto et al. (2017)
25^#^	WT	WT	Normal	3.3	Hz	Borsatto et al. (2017)
26^#^	WT	WT	Normal	4.1	Hz	Borsatto et al. (2014)
27^#^	WT	WT	Normal	3.7	Hz	Borsatto et al. (2014)
28^#^	c.1330G>C (p.Asp444His)	WT	Normal	3.5	Hz	In this study
29^#^	c.1368A>C (p.Gln456His)	WT	Normal	2.8	Hz	Borsatto et al. (2017)
30^#^	c.1330G>C (p.Asp444His)	c.1284C>T (p.Tyr428Tyr)	Normal	4.4	Hz	Borsatto et al. (2017)
31^#^	c.1330G>C (p.Asp444His)	WT	Normal	3.8	Hz	Borsatto et al. (2014)
32^#^	c.1330G>C (p.Asp444His)	WT	Normal	3.1	Hz	Borsatto et al. (2014)
33^#^	WT	c.1330G>C (p.Asp444His)	Normal	4.2	Hz	Borsatto et al. (2017)
34	c.1330G>C (p.Asp444His)	WT	Partial	2.1	Partial/Hz	Borsatto et al. (2017)
35	c.1368A>C (p.Gln456His)	WT	Partial	2.1	Partial/Hz	Borsatto et al. (2017)
36	c.[755A>G;1330G>C]^*^	p.Asp252Gly / p.Asp444His^*^	Partial	2.2	Partial/Hz	Borsatto et al. (2017)
37	c.1330G>C (p.Asp444His)	c.479G>A (p.Cys160Tyr)	Partial/Hz	2.3	Partial/Hz	In this study
38	c.1330G>C (p.Asp444His)	c.1330G>C (p.Asp444His)	Hz	2.2	Partial/Hz	Borsatto et al. (2017)
39	c.1330G>C (p.Asp444His)	c.1330G>C (p.Asp444His)	Hz	2.3	Partial/Hz	Borsatto et al. (2017)
40	c.1330G>C (p.Asp444His)	c.1330G>C (p.Asp444His)	Hz	2.2	Partial/Hz	Borsatto et al. (2017)
41	c.1330G>C (p.Asp444His)	c.1330G>C (p.Asp444His)	Hz	2.3	Partial/Hz	Borsatto et al. (2017)
42	c.1330G>C (p.Asp444His)	c.1330G>C (p.Asp444His)	Hz	2.3	Partial/Hz	In this study
43	c.278A>G (p.Tyr93Cys)	c.1330G>C (p.Asp444His)	Hz	2.1	Partial/Hz	In this study
44	c.1330G>C (p.Asp444His)	c.479G>A (p.Cys160Tyr)	Hz	2.3	Partial/Hz	Borsatto et al. (2014)
45	c.1330G>C (p.Asp444His)	c.1337T>C (p.Leu446Pro)	Unknown	2.2	Partial/Hz	Borsatto et al. (2017)
46	c.278A>G (p.Tyr93Cys)	WT	Unknown	2.3	Partial/Hz	In this study
47	c.278A>G (p.Tyr93Cys)	WT	Unknown	2.2	Partial/Hz	Borsatto et al. (2017)
48	c.278A>G (p.Tyr93Cys)	WT	Unknown	2.2	Partial/Hz	In this study
49	c.[595G>A;1330G>C;1629C>A]^*^	p.Val199Met / p.Asp444Hist / p.Cys471Cys^*^	Unknown	2.2	Partial/Hz	Borsatto et al. (2017)
50	WT	c.278A>G (p.Tyr93Cys)	Hz	2.2	Partial/Hz	Borsatto et al. (2017)
51	c.[755A>G;1330G>C]^*^	p.Asp252Gly / p.Asp444His^*^	Hz	2.2	Partial/Hz	Borsatto et al. (2017)
52	WT	c.1368A>C (p.Gln456His)	Hz	2.1	Partial/Hz	Borsatto et al. (2017)
53	WT	c.1330G>C (p.Asp444His)	Normal	4.9	Hz/Normal	Borsatto et al. (2014)
54	WT	c.1330G>C (p.Asp444His)	Normal	4.9	Hz/Normal	Borsatto et al. (2017)
55	WT	c.1330G>C (p.Asp444His)	Normal	4.9	Hz/Normal	Borsatto et al. (2017)
56	WT	c.1330G>C (p.Asp444His)	Normal	4.9	Hz/Normal	Borsatto et al. (2017)
57	WT	c.1330G>C (p.Asp444His)	Normal	4.9	Hz/Normal	Borsatto et al. (2017)
58	WT	c.1330G>C (p.Asp444His)	Normal	4.9	Hz/Normal	In this study
59	c.1330G>C (p.Asp444His)	WT	Normal	5.0	Hz/Normal	Borsatto et al. (2017)
60	c.1330G>C (p.Asp444His)	WT	Normal	5.0	Hz/Normal	In this study
61	c.1330G>C (p.Asp444His)	WT	Normal	5.0	Hz/Normal	In this study
62	WT	c.1629C>A (p.Cys471Cys)	Normal	4.9	Hz/Normal	Borsatto et al. (2014)
63	WT	c.1629C>A (p.Cys471Cys)	Normal	5.0	Hz/Normal	Borsatto et al. (2017)
64	c.1629C>A (p.Cys471Cys)	WT	Normal	4.9	Hz/Normal	Borsatto et al. (2017)
65	c.1629C>A (p.Cys471Cys)	WT	Normal	4.9	Hz/Normal	Borsatto et al. (2014)
66	c.1629C>A (p.Cys471Cys)	WT	Normal	4.9	Hz/Normal	In this study
67	c.1629C>A (p.Cys471Cys)	WT	Normal	4.9	Hz/Normal	In this study
68	WT	WT	Normal	5.0	Hz/Normal	Borsatto et al. (2017)
69	c.1330G>C (p.Asp444His)	c.119T>C (p.Leu40Pro)	Unknown	1.7	Partial	Borsatto et al. (2014)
70	c.1330G>C (p.Asp444His)	c.755A>G (p.Asp252Gly)	Partial	1.9	Partial	Borsatto et al. (2017)
71	c.1330G>C (p.Asp444His)	c.755A>G (p.Asp252Gly)	Partial	1.4	Partial	Borsatto et al. (2014)
72	c.1330G>C (p.Asp444His)	c.755A>G (p.Asp252Gly)	Partial	1.2	Partial	Borsatto et al. (2014)
73	c.1330G>C (p.Asp444His)	c.755A>G (p.Asp252Gly)	Partial	1.8	Partial	Borsatto et al. (2017)
74	c.755A>G (p.Asp252Gly)	c.1330G>C (p.Asp444His)	Partial	1.4	Partial	In this study
75	c.1330G>C (p.Asp444His)	c.[511G>A;1330G>C] (p.Ala171Thr / p.Asp444His)	Partial	1.4	Partial	Borsatto et al. (2014)
76	c.1330G>C (p.Asp444His)	c.[470G>A;1330G>C] ( p.Arg157His / p.Asp444His)	Partial	1.8	Partial	Borsatto et al. (2014)
77	c.1330G>C (p.Asp444His)	c.[470G>A;1330G>C] ( p.Arg157His / p.Asp444His)	Partial	1.9	Partial	Borsatto et al. (2017)
78	c.[1284C>T;1489C>T] (p.Tyr428Tyr / p.Pro497Ser)	c.1330G>C (p.Asp444His)	Partial	2.0	Partial	Borsatto et al. (2017)
79	c.1330G>C (p.Asp444His)	c.594_596del (p.Val199del)	Partial	1.9	Partial	Borsatto et al. (2014)
80	c.1330G>C (p.Asp444His)	c.594_596del (p.Val199del)	Partial	2.0	Partial	Borsatto et al. (2017)
81	c.1330G>C (p.Asp444His)	c.98_104del (fs)	Partial	1.5	Partial	Borsatto et al. (2014)
82	c.1330G>C (p.Asp444His)	c.98_104del (fs)	Partial	1.6	Partial	Borsatto et al. (2017)
83	c.[98_104del;1330G>C]^*^	p.Cys33fs / p.Asp444His^*^	Partial	2.0	Partial	Borsatto et al. (2017)
84	c.[100G>A;1330G>C]^*^	p.Gly34Ser / p.Asp444His^*^	Partial / Hz	2.04	Partial	Borsatto et al. (2014)
85	c.1368A>C (p.Gln456His)	c.1330G>C (p.Asp444His)	Partial	2.0	Partial	Borsatto et al. (2017)
86^#^	WT	c.1330G>C (p.Asp444His)	Normal	1.2	Partial	Borsatto et al. (2017)
87^#^	WT	c.1330G>C (p.Asp444His)	Normal	1.2	Partial	Borsatto et al. (2017)
88	c.[1330G>C;1629C>A] (p.Asp444His / p.Ala171Thr)	c.1466A>G (p.Asn489Ser)	Unknown	1.4	Partial	Borsatto et al. (2017)
89	c.643C>T (p.Leu215Phe)	c.755A>G (p.Asp252Gly)	Total	0.04	Total	Borsatto et al. (2014)
90	c.755A>G (p.Asp252Gly)	c.755A>G (p.Asp252Gly)	Total	0.44	Total	Borsatto et al. (2014)
91	c.1227_1241del (p.Trp409fs)	c.1227_1241del (p.Trp409fs)	Total	0.09	Total	Borsatto et al. (2017)
92	c.1612C>T (p.Arg538Cys)	c.1612C>T (p.Arg538Cys)	Total	0.12	Total	Borsatto et al. (2014)

BD = biotinidase deficiency WT - Wild Type fs = frameshift.

Normal reference range of the enzyme: 5.0±10 nmol/min/mL. The biochemical phenotype among patients who presented activity lower than 5.0 nmol/min/mL: <0.75 (<10%), profound BD; 0.75±2.25 (10±30%), partial BD; and 2.26±4.99 (30.1±66.5%), heterozygous activity.

* = Whether it is in cis or trans configuration with the other variant found remains undetermined.

# = Patient with discrepancies between Expected BD according to genotype and Type of BD according to enzyme activity

For genomic DNA extraction, blood samples were collected in EDTA-containing tubes and processed using the Easy-DNA gDNA Purification kit (Thermo Fisher). The 3′UTR of the *BTD* gene was amplified by PCR with specific primers. The products were purified with 20% PEG 8000/2.5M NaCl and sequenced by Sanger method. Sequences were analyzed in the Chromas Lite software and aligned with the reference sequence NG_008019.1 in Blast/NCBI. 

### *In silico* analysis

Variants were searched in the 3′UTR available in worldwide public genomic databases: LOVD (Fokkema et al., 2011 - 515,500 variants in 162,000 patients), gnomAD (Karczewski et al., 2020 - 76,156 genomes and 125,748 exomes), Online Archive of Brazilian Mutations - AbraOM (Naslavsky et al., 2017 - 1,171 genomes and 609 exomes) and Varsome clinical platform (Zhang et al., 2020 - 70 public genomic databases). Variants with rs snp code were classified according to the ACMG classification (Richards et al., 2015).

To evaluate conservation of the 3′UTR of *BTD*, sequence alignments between different species were performed in MEGA software (version 7.0.26, Kumar et al., 2016), using the ClustalW algorithm (version 2.1, Thompson et al., 1994). Evolutionarily conserved regions were mapped in ECR Browser (Ovcharenko et al., 2004). The chromosomal position provided in the Atlas of UTR Regulatory Activity (AURA) (Dassi et al., 2014) was used to locate the 3′UTR of *BTD* gene.

To investigate miRNAs that might regulate *BTD* expression, mirBase (Kozomara and Griffiths-Jones, 2014) miRTarBase (Huang et al., 2019), TarBase (Karagkouni et al., 2017), TargetScanHuman (Agarwal et al., 2015), miRWalk (Dweep et al., 2011), and miRGate software (Andrés-León et al., 2015) were used. To explore the shared miRNAs across the biotin metabolism related genes, the TopCluster web service (Kaimal et al., 2010) was used.

The miRanda (Enright et al., 2003), mirSVR (Betel et al., 2010), and microRNA.org (Betel *et al.*, 2008) algorithms were used for analysis of miRNAs target sites associated with *BTD*. The cutoff points for this analysis were a binding free energy of -25 Kcal - proposed as more stable by Seffens and Digby (1999), and the search for evolutionarily conserved targets (mainly 8-mer), as suggested by Garcia *et al.* (2011). 

For polyadenylation analysis, the constitutive site was characterized according to the reference sequence curated by NCBI. APADB (Müller et al., 2014), APASDB (You et al., 2015) databases and the PolyA_SVM (Structural Support Vector Machine) algorithm of the RegRNA package (v. 2.0, Chang et al., 2013) were used to quantify sites usage and polyadenylation signals.

To identify other regulatory elements in the 3′UTR, the software RegRNA v. 2.0 (Chang et al., 2013) and ARE Site 2 (Fallmann et al., 2015) were used. Secondary structures formed by miRNA-3′UTR interactions were obtained through the RNAfold Web server (Gruber et al., 2008).

## Results

No variant was identified in the analysis of the 3′UTR of the *BTD* gene. 

### *In silico* analysis

Conservation analysis showed that the 3′UTR of the *BTD* gene is highly conserved in primates. Alignments between the human *vs.* rat, mouse, cow, dog, rhesus monkey and chimpanzee 3′UTR sequences of *BTD* gene revealed identities of 73.1%, 71.6%, 70.6%, 72.1%, 94%, and 99% respectively.

In the search of variants in genomic public databases, 43 variants were found in the AbraOM, 32 of them predicted as ‘variant of uncertain significance’ (VUS), and 11 as ‘benign’. In the gnomAD, nine variants were found, all predicted by the ACMG as ‘VUS’. In the LOVD database, three variants were found - one predicted as ‘VUS’ and two as ‘benign’. The allele frequencies and the respective rsSNP as shown in Table 2. 

**Table 2 -:** 3’UTR variant frequencies in Brazilian genomic databases (ABraOM) and worldwide databases (gnomAD and LOVD).

Database	Variant	rsSNP code	Prediction	Allele Frequency
ABraOM	c.^*^83A>T	rs151091741	Benign	0.016652
	c.^*^96G>A	rs530884413	VUS	0.000427
	c.^*^211G>A	rs78601074	VUS	0.002989
	c.^*^251T>G	rs973865557	VUS	0.000427
	c.^*^276C>T	rs529324919	VUS	0.001708
	c.^*^310A>G	rs189885639	VUS	0.003843
	c.^*^348G>T	rs187175217	VUS	0.007669
	c.^*^366A>T	rs1004621476	VUS	0.026046
	c.^*^368C>T	rs1034718749	VUS	0.013237
	c.^*^371G>T	rs960652511	VUS	0.017079
	c.^*^452A>G	rs79151199	VUS	0.002989
	c.^*^471G>T	rs115371875	VUS	0.005124
	c.^*^537C>T	rs180874910	VUS	0.005978
	c.^*^734A>G	rs1019755479	VUS	0.000854
	c.^*^748G>C	rs965102987	VUS	0.000427
	c.^*^549C>T	rs572632251	VUS	0.000854
	c.^*^768C>T	rs73150121	VUS	0.002989
	c.^*^573G>A	rs965394624	VUS	0.000427
	c.^*^811G>A	rs559860346	VUS	0.000854
	c.^*^847T>A	rs9647358	Benign	0.16567
	c.^*^916G>A	rs1009938115	VUS	0.000854
	c.^*^903G>A	rs57114474	Benign	0.094791
	c.^*^983T>C	rs76866504	Benign	0.015371
	c.^*^1009A>G	rs771654037	VUS	0.000854
	c.^*^1021C>T	rs772800231	VUS	0.000854
	c.^*^1142G>A	rs575407757	VUS	0.000427
	c.^*^1337C>T	rs55866239	Benign	0.05807
	c.^*^1461G>T	rs972571533	VUS	0.000427
	c.^*^1501C>T	rs117876477	VUS	0.002989
	c.^*^1546T>C	rs1041474484	VUS	0.000427
	c.^*^1059A>G	rs558313573	VUS	0.000854
	c.^*^1652C>T	rs3796305	Benign	0.041418
	c.^*^1678C>T	rs1027781482	VUS	0.000854
	c.^*^1686C>T	rs145664140	VUS	0.002135
	c.^*^1693C>T	rs2455852	Benign	0.535013
	c.^*^1707G>A	rs1017619524	VUS	0.000427
	c.^*^1763C>T	rs2470530	Benign	0.686166
	c.^*^1799G>A	rs3796302	Benign	0.094791
	c.^*^1949C>T	rs1017670214	VUS	0.000427
	c.^*^2063C>T	rs73145546	Benign	0.026046
	c.^*^2106G>A	rs915646184	VUS	0.000427
	c.^*^2121C>T	rs77633353	VUS	0.002135
	c.^*^2123G>A	rs2470531	Benign	0.532878
gnomAD	c.^*^8G>A	rs773652007	VUS	0.000037
	c.^*^15C>T	rs763033233	VUS	0.000103
	c.^*^23C>T	rs766374135	VUS	0.000038
	c.^*^24G>A	rs374047871	VUS	0.000080
	c.^*^29C>T	rs1344267775	VUS	0.000008
	c.^*^32G>T	rs1200505812	VUS	0.000004
	c.^*^43G>T	rs200147547	VUS	0.000030
	c.^*^53C>T	rs761431603	VUS	0.000063
	c.^*^54A>C	rs1404681940	VUS	0.000031
LOVD	c.^*^211G>A	rs78601074	VUS	0.000358
	c.^*^847T>A	rs9647358	Benign	0.2132
	c.^*^2123G>A	rs2470531	Benign	0.5559

*In silico* analysis of miRNAs yielded highly variable results. The number of miRNAs predicted in *BTD* gene were: 51 in miRGate database, 35 in miRTarBase, 5 in miRWalk, 4 in TarBase and 2 in TargetScanHuman (Table 3).

**Table 3 -:** miRNAs associated with the *BTD* gene in different search methods and databases.

miRGate	miRTarBase	miRWalk	TarBase	TargetScan
hsa-mir-1227-3p	hsa-miR-10b-3p	hsa-miR-3620-3p	hsa-miR-129-2-3p	hsa-miR-145-5p
hsa-mir-1233-5p	hsa-miR-1247-3p	hsa-miR-4743-3p	hsa-miR-200b-3p	hsa-miR-5195-3p
hsa-mir-1266-5p	hsa-miR-1267	hsa-miR-6499-3p	hsa-miR-21-3p	
hsa-mir-1910-3p	hsa-miR-219b-3p	hsa-miR-6808-5p	hsa-miR-7-5p	
hsa-mir-3127-5p	hsa-miR-30d-3p	hsa-miR-6837-3p		
hsa-mir-3137	hsa-miR-30e-3p			
hsa-mir-3158-3p	hsa-miR-340-5p			
hsa-mir-3190-3p	hsa-miR-3620-3p			
hsa-mir-3190-5p	hsa-miR-367-5p			
hsa-mir-363-5p	hsa-miR-3929			
hsa-mir-3666	hsa-miR-3942-3p			
hsa-mir-4323	hsa-miR-4257			
hsa-mir-4417	hsa-miR-4419b			
hsa-mir-4435	hsa-miR-4478			
hsa-mir-4446-3p	hsa-miR-4649-3p			
hsa-mir-4449	hsa-miR-4652-3p			
hsa-mir-4518	hsa-miR-4670-3p			
hsa-mir-4640-3p	hsa-miR-4722-5p			
hsa-mir-4647	hsa-miR-4729			
hsa-mir-4657	hsa-miR-4743-3p			
hsa-mir-4674	hsa-miR-4768-3p			
hsa-mir-4685-5p	hsa-miR-5100			
hsa-mir-4708-3p	hsa-miR-5584-3p			
hsa-mir-4737	hsa-miR-5696			
hsa-mir-4741	hsa-miR-570-3p			
hsa-mir-4758-3p	hsa-miR-579-3p			
hsa-mir-485-5p	hsa-miR-6125			
hsa-mir-5001-3p	hsa-miR-6499-3p			
hsa-mir-5007-5p	hsa-miR-6516-5p			
hsa-mir-505-3p	hsa-miR-664a-3p			
hsa-mir-548q	hsa-miR-664b-3p			
hsa-mir-603	hsa-miR-6808-5p			
hsa-mir-6511a-5p	hsa-miR-6893-5p			
hsa-mir-6745	hsa-miR-7160-5p			
hsa-mir-6756-5p	hsa-miR-940			
hsa-mir-6764-5p				
hsa-mir-6766-5p				
hsa-mir-6798-3p				
hsa-mir-6808-5p				
hsa-mir-6811-3p				
hsa-mir-6823-5p				
hsa-mir-6833-5p				
hsa-mir-6834-5p				
hsa-mir-6837-5p				
hsa-mir-6873-5p				
hsa-mir-6882-3p				
hsa-mir-6884-5p				
hsa-mir-7114-5p				
hsa-mir-718				
hsa-mir-874-5p				
hsa-mir-938				

**Figure 2 -:**
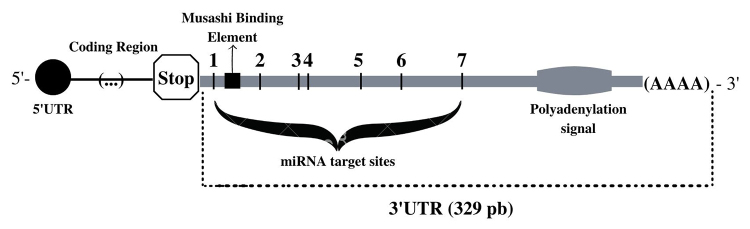
Summary of the elements found associated with the 3’UTR of *BTD*.

Seven miRNA target sites (Table 4) and one RNA binding protein (Musashi Binding Element) were identified. The mapped elements were presented in Figure 2.

**Table 4 -:** Prediction of miRNA target sites in *BTD* according to mirSVR and TargetScanHuman algorithms.

miRNA ID	mirSVR score	Phast Cons score	Type seed	Reference
hsa-miR-6764-5p	0.12	0.55	7mer-m8 (1) 7mer-A1 (1)	Pathak *et al.* (2017)
hsa-miR-8066	-1.29	0.52	7mer-A1 (1)	Wang *et al.* (2013)
hsa-miR-940	-0.01	0.44	8mer (1) 6mer (1)	Rajendiran *et al.* (2014)
hsa-miR-1267	-0.39	0.52	7mer-m8 (2)	Tomasetti *et al.* (2016)
hsa-miR-5195-3p	-0.08	0.43	8mer (2) 6mer (1)	Salehi *et al.* (2017)
hsa-miR-34a-5p	-0.01	0.44	7mer-m8 (1)	Kálmán *et al.* (2014)
hsa-miR-1915-3p	-0.80	0.49	7mer-m8 (1)	Migita *et al.* (2003)

mirSVR and Phast Cons score are related to conservation between the seed region of the miRNA and its target gene. The number in parentheses indicates how many sites of mRNA pairing:miRNA the detected algorithm.

Forty-nine miRNAs were associated with genes that interact with the *BTD* gene in biotin metabolism (Table 5). The only miRNA shared between *BTD* and *HLCS* was the hsa-miR-222.

**Table 5 -:** Genes involved in biotin metabolism and number of miRNAs predicted to influence the metabolic pathways of alanine, biotin, citrate and pyruvate.

Gene	Location	Name	miRNAs	Metabolism
*HLCS*	21q22.1	Holocarboxylase Syntetase	8	Biotin
*MCCC1*	3q27	Methylcrotonoyl- Coenzyme A carboxylase 1 (alfa)	6	Biotin
*PC*	11q13.4	Pyruvate Carboxylase	14	Alanine, Biotin, Cytrate and Pyruvate
*SPCS1*	3p21.1	Signal Peptidase Complex subunit 1	10	Biotin
*SPCS3*	4q34.2	Signal Peptidase Complex subunit 3	11	Biotin

**Figure 3 -:**
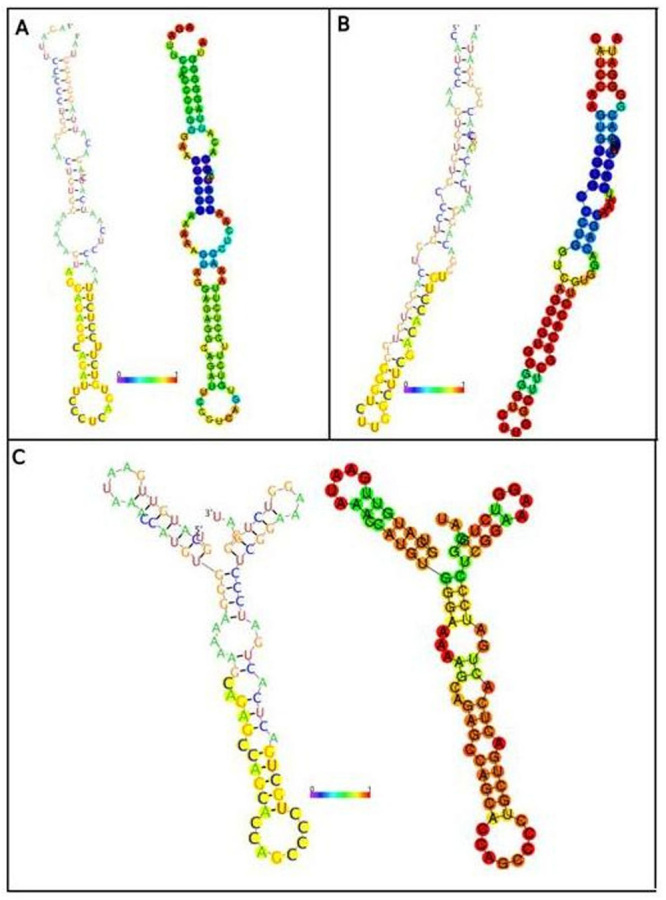
Secondary structures of the miRNAs. A: miRNA hsa-miR-3916. B: miRNA hsa-miR-3934. C: miRNA hsa-miR-4763-5p. The yellow region shows the mature miRNA and the likelihood of them being associated with the *BTD* gene. The red color corresponds to the highest correlation between free energy binding between miRNA: mRNA and its interaction.

The three best-predicted secondary structure models are presented in Figure 3. The most appropriate secondary structure according to RNAfold analysis was the model of interaction between the 3′UTR of the *BTD* gene and hsa-miR-3934, with a binding free energy of -25.35 Kcal. 

The polyadenylation signal used by the *BTD* gene coincides with the canonical AAUAAA hexamer. The dinucleotide that identifies the cleavage site was AA. Results from the APASdb database and the PolyA_SVM algorithm showed that the *BTD* gene has two major mapped polyadenylation sites. The first signal begins at position 2044 and has 32 pb; the second signal begins at position 2329 and has 32 pb. According to the APADB database, both polyadenylation sites of the *BTD* gene are located in the 3′UTR at positions chr3:15687323 (86.1% of usage) and chr3:15683749 (11.4% of usage). 

Six AU-rich elements were identified: TTTTT, ATTTA, ATTTT, TTTTA, TATTTTA and AATAAA.

## Discussion

In this study, we investigated the presence of variants in the 3′UTR of the *BTD* gene in individuals with reduced biotinidase activity and, using bioinformatics tools, we discussed a possible relationship by regulatory elements with the expression of the *BTD* gene.

As far as we know, the 3′UTR had never been characterized in patients. As observed in the present cohort about 20% of the patients have discrepancies between expected BD according to genotype and type of BD according to enzyme activity.

The hypothesis for this investigation came from other diseases that present phenotype modification due to variants in the 3’UTR of the affected gene, as Glycogen Storage Disease Ia (Karthi et al., 2017) and Haemophilia A (Rosset et al., 2016). Modified regulatory elements may affect the interaction of the UTRs with proteins and microRNAs causing modulation of mRNA transcription, secondary structure, stability, localization, translation, and access to regulators like microRNAs (miRNAs), RNA‐binding proteins (RBPs) and justify the discrepancies between genotype and phenotype (Steri et al., 2018; Skarp et al., 2020). 

The high conservation of the 3’UTR was observed among the 92 patients analyzed proved by the 100% homology - no variants were found. Variant databases reinforced the conservation of the region through low frequencies of variants. 

Subsequent investigations of the 3’UTR found several miRNAs and elements present in the region. Variations present in patients could justify differences in gene expression through factors related to 3’UTR.

The main predicted miRNAs associated with the *BTD* gene were: hsa-miR-7-5p, previously implicated in suppression of cell proliferation, induction of apoptosis, and angiogenesis (Li et al., 2016; Luo et al., 2018); hsa-miR-34a-5p, which is involved in cell proliferation and an important regulator of the central nervous system (Agostini and Knight, 2014; Jauhari and Yadav, 2019); and hsa-miR-145, identified in neonates and expressed specifically in the liver, where biotinidase expression is also higher (Fu et al., 2005; Noh et al., 2013).

The miR-7 cluster is known to be associated with genes related to the nervous system. Dostie et al. (2003) demonstrated that this miRNA may be unregulated in neuronal cells in spinal muscular atrophy, and involved in the neurological dysfunctions associated with Waisman Syndrome and Fragile X Syndrome. Untreated BD may lead to neurological problems and developmental delay. Thus, it is important to note that this miRNA, along with several potentially related factors, may be a candidate for investigation.

Hearing loss is a common sensorineural impairment in general populations. Experiments done in the inner ear of mice and humans have found differential expression of five miRNAs, among them miR-30, associated with different stages of ear development (Rudnicki and Avraham, 2012). In the present analysis, miR-30 was associated with the *BTD* gene. Among patients with total BD, 75% of affected children have hearing loss (Wolf et al., 2002), with variable but usually irreversible severity. 

Forty-nine miRNAs associated with genes that interact with the *BTD* were identified in the biotin metabolic pathway. These miRNAs have already been implicated in cell signaling, glycosylation pathways, and in arginine, biotin, tyrosine, and thiamine metabolism (Ortega et al., 2010). The *PC* gene that encodes pyruvate carboxylase, a biotin-dependent carboxylase, was found not only in the biotin metabolic pathway but also in alanine, citrate cycle, and pyruvate metabolic pathways (Rottiers and Näär, 2012).

Gene ontology analysis showed that these genes are involved in several biological processes, and act as coenzymes and in the metabolism of small molecules (Gene ontology: Fisher’s exact with FDR multiple test correction: 9.95e-20 / 1.55e-15) (Thomas et al., 2003; Mi et al., 2013).

Among the most prominent results is the *HLCS* target gene. *HLCS* encodes the holocarboxylase synthetase that activates biotin-dependent carboxylases and catalyzes the binding of biotin to biotinidase. Experiments have shown that miR-539 decreases holocarboxylase synthetase levels, with the abundance of miR-539 being significantly higher at physiological biotin concentrations than in biotin-deficient and biotin-supplemented media, in all cell lines tested (Segura et al., 2013). The results of this study suggest that miR-539 may be one of several factors that detect biotin and regulate holocarboxylase synthetase levels. In the present study, this miRNA was not directly associated to the *BTD* gene, but to the holocarboxylase synthetase gene *HLCS*.

The *SPCS1* and *SPCS3* genes - subunits of the peptidase signal complex that act as hydrolases and participate in degradation of lysine (Kailes and Hartmann, 1996) - also stood out. The lysine present in the biotin-lysine complex (biocytin) is believed to be degraded through the action of this complex. The miRNAs associated with these genes may have an impact on expression of *SPCS1* and *SPCS3* and, consequently, on lysine degradation, preventing biotin recycled into its free form. In addition, hsa-miR-204 and hsa-miR-211, both predicted to be associated with *SPCS1,* are implicated in mechanisms of cell proliferation and metastasis in several types of cancer, including breast, colon, and lung cancer (Mazar et al., 2010).

Esau et al. (2006) found that miR-122 allows the liver to function properly in adult mice. This miRNA is an important mechanism for regulation of genes involved in hepatic lipid metabolism. This corroborates the findings of Saha and Ruderman (2003) that observed negative effects on mice lipogenesis whereby a reduction in *ACC* gene expression, particularly *ACC2*, led to a decrease in malonyl CoA and subsequent increase in fatty acid oxidation. As biotinidase acts as a cofactor for several carboxylases, miRNAs may be involved in feedback regulation of this system. This miRNA was not found to be associated with *BTD*, but appears to be involved with citrate and pyruvate metabolism genes. 

Based on the assumption that a single miRNA can regulate several target genes, miR-31-3p and miR-34a-5p were associated with the *BTD* gene and with the *PCCA* and *PCCB* genes*,* which encode subunits of the enzyme propionyl-coA-carboxylase, one of the biotin-dependent carboxylases. Dysfunction in these genes can lead to propionic acidemia, a disease characterized mainly by neurological and cardiac damage. Rivera-Barahona et al. (2017) found that these miRNAs are deregulated in the liver of mice; more specifically, overexpression of the miR-34 family is observed in patients with cardiac involvement, and is associated with other neurodegenerative diseases. 

## Conclusions

The present study was pioneer in the analysis of the 3′UTR of *BTD* gene in individuals with reduced biotinidase activity. Although the sequencing of this region has not found variants, it described their evolutive conservation. 

The study of the 3′UTR in individuals with reduced biotinidase activity allowed us to conclude that variants in this region do not explain the genotype-phenotype discrepancies found in Brazilian patients. However, several factors as miRNAs sites and regulatory elements have been identified, which may influence the expression patterns of the *BTD* gene. To date, there are no strongly validated interactions between miRNAs and the *BTD* gene. Thus, its experimental validation remains as a perspective for future research. 
